# Alzheimer’s Disease Enhanced Tonic Inhibition is Correlated With Upregulated Astrocyte GABA Transporter-3/4 in a Knock-In *APP* Mouse Model

**DOI:** 10.3389/fphar.2022.822499

**Published:** 2022-02-03

**Authors:** Yousif Aldabbagh, Anam Islam, Weicong Zhang, Paul Whiting, Afia B. Ali

**Affiliations:** ^1^ UCL School of Pharmacy, London, United Kingdom; ^2^ Alzheimer’s Research UK Drug Discovery Institute, Queen Square Institute of Neurology, London, United Kingdom

**Keywords:** alzheheimer’s disease, hippocampus, GABA, excitation, astrocytes, amyloid-β, dentate gyrus

## Abstract

Cognitive decline is a major symptom in Alzheimer’s disease (AD), which is strongly associated with synaptic excitatory-inhibitory imbalance. Here, we investigated whether astrocyte-specific GABA transporter 3/4 (GAT3/4) is altered in *APP* knock-in mouse model of AD and whether this is correlated with changes in principal cell excitability. Using the *APP* ^*NL-F/NL-F*^ knock-in mouse model of AD, aged-matched to wild-type mice, we performed *in vitro* electrophysiological whole-cell recordings combined with immunohistochemistry in the CA1 and dentate gyrus (DG) regions of the hippocampus. We observed a higher expression of GAD67, an enzyme that catalyses GABA production, and GAT3/4 in reactive astrocytes labelled with GFAP, which correlated with an enhanced tonic inhibition in the CA1 and DG of 12–16 month-old *APP* ^*NL-F/NL-F*^ mice compared to the age-matched wild-type animals. Comparative neuroanatomy experiments performed using post-mortem brain tissue from human AD patients, age-matched to healthy controls, mirrored the results obtained using mice tissue. Blocking GAT3/4 associated tonic inhibition recorded in CA1 and DG principal cells resulted in an increased membrane input resistance, enhanced firing frequency and synaptic excitation in both wild-type and *APP* ^*NL-F/NL-F*^ mice. These effects exacerbated synaptic hyperactivity reported previously in the *APP* ^*NL-F/NL-F*^ mice model. Our data suggest that an alteration in astrocyte GABA homeostasis is correlated with increased tonic inhibition in the hippocampus, which probably plays an important compensatory role in restoring AD-associated synaptic hyperactivity. Therefore, reducing tonic inhibition through GAT3/4 may not be a good therapeutic strategy for AD

## 1 Introduction

Alzheimer’s disease (AD) is the most prevalent cause of dementia in the elderly, affecting 6–8% of the world’s population over 65 years old (WHO, 2017). AD is a progressive disorder that leads to cognitive deficits which severely reduce quality of life (Burns and Iliffe, 2009), and is described macroscopically by loss of brain volume and shrinkage of cortical gyri, with the entorhinal cortex and hippocampus being heavily affected (Stelzma et al., 1995).

The major pathological hallmarks of AD are neuroinflammation, presence of severe neuropathological lesions, including amyloid-*β* (A*β*) plaques, synaptic loss, and neuronal death. One of the key cell types that modulate the neuroinflammatory response in AD are astrocytes, the most abundant glial cells in the brain, supported by glial fibrillary acidic protein (GFAP). A*β* plaques have been shown to localise and trigger the activation of astrocytes (Akiyama, 2000) and it is well established that prolonged neuroinflammation maintained by reactive astrocytes can have catastrophic effects on the neuronal environment and potentiate neurodegeneration in AD (Medeiros and LaFerla, 2013). Recent studies have shown that A*β*-induced dysfunction of astrocytes networks can lead to dysregulated neuronal networks and a positive feedback loop with A*β* formation and deposition; with similar effects not seen in the non-pathological ageing brain (Gómez-Gonzalo et al., 2017; Lines et al., 2022)

Astrocytes also play a key role in the ‘tripartite’ synapse where the perisynaptic astrocytic processes envelop the pre- and postsynaptic elements of neurons; consequently, maintaining neuronal homeostasis by the uptake of excess neurotransmitters or release of gliotransmitters such as *?*-aminobutyric acid (GABA), thus modulating synaptic signalling (Parpura et al., 1994; Delekate et al., 2014; Jo et al., 2014; Ishibashi et al., 2019; Patel et al., 2019). For example, under “normal” physiological conditions, astrocytes uptake excessive neuronally released glutamate from the synapse through Na^+^-coupled excitatory amino acid transporters (EAAT1/EAAT2) (Lehre and Danbolt, 1998; Kojima et al., 1999), and GABA through astrocyte-specific GABA transporters, GAT3/4 (Liu et al., 1993), abundantly found on astrocytic processes (Ribak et al., 1996; Conti et al., 2004), which are thought to be crucial for setting the level of background tonic inhibition. However, these mechanisms seem to alter during pathological conditions, which can lead to reversal of such transporters and extrusion of GABA into the synaptic space, ultimately impacting the fine-tuning of inhibitory synapses (Conti et al., 2004; Héja et al., 2012). Interestingly, following subcortical stroke an enhanced tonic inhibition, via astrocytes was shown to induce neuronal glucose hypometabolism, impairing functional recovery and neuronal activity (Nam et al., 2020). Similarly, evidence suggests that elevated levels of tonic inhibition in the dentate gyrus (DG), impaired synaptic plasticity as well as memory function via GAT3/4 in a 5xFAD mouse model (Wu et al., 2014). These findings have huge relevance in understanding the role of astrocytes in maintaining synaptic homeostasis in altered physiological states such as AD-associated synaptic hyperexcitability, a phenotype that spans from human studies to animals, and iPSC cell models (Hazra et al., 2013; Busche and Konnerth, 2016; Ghatak et al., 2019; Petrache et al., 2019). AD-associated hyperexcitability is thought to spread the disease pathology between brain subregions (Khan et al., 2014; Petrache et al., 2019). The process by which this develops remains to be fully answered, with one hypothesis referring to the selective aberrant behaviour of inhibitory neurotransmitter GABA-containing interneurons (Rice et al., 2020; Shi et al., 2020; Xu et al., 2020; Reid et al., 2021), despite an elevated background inhibition observed in AD by others (Jo et al., 2014; Wu et al., 2014). Surprisingly, the levels of astrocyte glutamate decarboxylase 67 (GAD67), an enzyme that converts glutamate to GABA, is shown to be elevated in astrocytes in AD patients and mouse models of AD (Wu et al., 2014). This suggests an altered role of astrocytes in GABA homeostasis via GAT3/4, which is thought to be related to the modified inhibitory tone of local neuronal circuits (Lee et al., 2011). Therefore, in this study, we investigated whether elevated GAD67 and GAT3/4 levels are associated with a higher background inhibitory tone and whether this correlated with intrinsic and synaptic excitability of hippocampal principal cells in a knock-in mouse model of AD.

## 2 Materials and Methods

### 2.1 Mouse Animal Procedures

All the procedures in this study were carried out in accordance with the British Home Office regulations under the Animal Scientific Procedure Act 1986, under the project licence PPL: P1ADA633A held by the principal investigator, Dr Afia Ali. All procedures were approved by both internal and external UCL ethics committees, and in accordance with the ARRIVE guidelines for reporting experiments involving animals (McGrath et al., 2010). A total of 78 animals (disease model and wild-type) were used in this study. The animals had *ad-libitum* access to food and water and were reared in cages of maximum of 5 inhabitants, with a day: night cycle of 12 h: 12 h.

The knock-in *APP* ^*NL-F/NL-F*^ AD mouse model was used for this study (Saito et al., 2014). This mouse model was chosen because it faithfully reproduces the effect of AD Aβ pathology without overexpression artefacts in a time-dependent manner. The *APP* ^*NL-F/NL-F*^ model has two familial AD (FAD) mutations: KM670/671NL (Swedish) and I716F (Iberian). The former increases *ß*-site cleavage of APP to produce elevated amounts of both A*β*40 and A*β*42, whereas the latter promotes γ-site cleavage at C-terminal position 42, thereby increasing the A*β*42/Aβ40 ratio in favour of the more hydrophobic A*β4*2 as seen in clinical AD. Thus, the *APP* ^*NL-F/NL-F*^ mouse model shows A*β* accumulation and related pathology in an age-dependent manner, with initial accumulation shown at 6 months (Saito et al., 2014). The knock-in line was crossed with C57BL/6 mice and the resulting heterozygous pairs were used for breeding but excluded from experiments. Only male *APP* ^*NL-F/NL-F*^ and age-matched wild-type (C57BL/6) mice, aged between 12 and 16 months were included.

Animals were genotyped via standard polymerase chain reaction using the following four primers: 5′-ATC​TCG​GAA​GTG​AAG​ATG-3′, 5′-TGT​AGA​TGA​GAA​CTT​AAC-3′, 5′-ATC​TCG​GAA​GTG​AAT​CTA-3′, and 5′-CGT​ATA​ATG​TAT​GCT​ATA​CGA​AG-3′ as previously described (Saito et al., 2014).

### 2.2 Tissue Collection and Preparation

#### 2.2.1 Mouse Brain Tissue

Tissue preparation was carried out as previously described (Petrache et al., 2019; Shi et al., 2020). All experiments were performed single-blinded. Mice were deeply anaesthetized using inhalation of isoflurane 4% followed by intraperitoneal injection of 60 mg/kg phenobarbitone. The level of anaesthesia was monitored using pedal and tail pinch reflexes, rate, depth, and pattern of respiration through observation and the colour of mucous membranes and skin. The mice were then perfused transcardially with artificial cerebrospinal fluid (ACSF) containing (in mM) 248 sucrose, 3.3 KCl, 1.4 NaH2PO4, 2.5 CaCl2, 1.2 MgCl2, 25.5 NaHCO3, and 15 glucose, bubbled with 95% O_2_ and 5% CO_2_. The animals were then decapitated, the brain removed and 300 µm thick coronal sections of the cortex and hippocampus were cut in ice-cold standard ACSF using an automated vibratome (Leica, Germany). This standard ACSF contained (in mM): 121 NaCl, 2.5 KCl, 1.3 NaH2PO_4_, two CaCl_2_, one MgCl_2_, 20 glucose and 26 NaHCO_3_, equilibrated with 95% O_2_ and 5% CO_2_ (pH, 7.3, osmolarity, 300–310 m Osm).

The brain slices were then incubated in ACSF for 30 min at 37°C and transferred to room temperature prior to recording. Brain slices were placed in a submerged chamber and superfused with ACSF at a rate of 1–2 ml/min for electrophysiological recordings.

For neuroanatomical studies, one-half of the brains were immediately fixed after perfusion in 4% paraformaldehyde and 0.1% glutaraldehyde in 0.1M phosphate buffer for 24 h prior to sectioning.

### 2.3 Human Brain Tissue

A total of 27 hippocampal post-mortem brain tissue sections from 13 AD patients and 14 age-matched control individuals were obtained from Queen Square Brain Bank for Neurological Disorders, UCL Institute of Neurology, according to the Human Tissue Act (HTA) 2004 and under the HTA license. Ethical approval was obtained from the local research ethics committee for the national hospital for Neurology and Neurosurgery. The information for cases used throughout is detailed in Table 1.

**TABLE 1:** *Patient demographic details of human cases used for neuroanatomy experiments*.

Cases ID	Group	Regions Used	Age (years)	Sex	Post-mortem Delay (hours)	Brain Weight (g)	Braak Staging	CERAD Score	Thal Staging
1	AD	CA1	67	Male	35.27	1,223	Braak 6	CERAD 3	Thal 5
2	AD	CA1	55	Female	47.50	1,100	Braak 6	Frequent	—
3	AD	CA1	90	Male	89	1,200	Braak 4	CERAD 0	Thal 1
4	AD	CA1	86	Male	96.1	1,203	Braak 6	CERAD 3	Thal 5
5	AD	CA1	68	Male	70.05	1,522	Braak 6	—	Thal 5
6	AD	CA1, DG	69	Male	35.04	891	Braak 6	Frequent	Thal 5
7	AD	CA1, DG	88	Male	58.1	1,084	Braak 6	—	Thal 5
8	AD	CA1, DG	70	Male	60.25	1,224	—-	—	—
9	AD	CA1, DG	62	Female	76.20	996	Braak 6	Frequent	Thal 5
10	AD	DG	63	Male	31.42	1,042	Braak 6	CERAD 3	Thal 5
11	AD	DG	64	Male	95.5	1,280	Braak 6	—	Thal 5
12	AD	DG	65	Male	34.25	1,089	Braak 5 or 6	C3	A3
13	AD	DG	79	Male	61.19	1,423	Braak 5 or 6	C3	A3
14	Control	CA1	101	Male	60.35	1,450	Braak 1	CERAD 0	—
15	Control	CA1	79	Male	105.5	1,355	Braak 2	—	—
16	Control	CA1	88	Male	96	1,240	Braak 2	CERAD 1	Thal 3
17	Control	CA1	71	Female	76	1,214	Braak 3	CERAD 2	Thal 2
18	Control	CA1	86	Female	120	1,234	Braak 2	—	—
19	Control	CA1	80	Female	49.10	1,242	Braak 2	—	—
20	Control	CA1	83	Male	105.00	1,244	Braak 4	CERAD 2	Thal 3
21	Control	CA1, DG	94	Female	89.25	1,541	Braak 3	Sparse	—
22	Control	CA1, DG	88	Male	27.30	1,300	Braak 4	—	Thal 3
23	Control	DG	76	Male	79	1,366	Braak 2	Cerad 0	Thal 1
24	Control	DG	80	Female	53	1,130	Braak 2	Cerad 0	Thal 3
25	Control	DG	84	Female	53	1,283	Braak 2	—	Thal 3
26	Control	DG	90	Male	46	1,213	Braak 4	Cerad 2	Thal 3
27	Control	DG	96	Female	60	1,032	Braak 2	—	Thal 2

### 2.4 Electrophysiology

Whole-cell somatic recordings were performed using patch electrodes with resistances of 8–11 MΩ made from filamented borosilicate glass capillaries (Harvard Apparatus, UK) and filled with a solution containing (in mM): 134 K gluconate, 10 HEPES, 10 phosphocreatine, two Na2ATP, 0.2 Na2GTP, and 0.2% w/v biocytin (pH, 7.3, osmolarity, 300–310 mOsm). Excitatory CA1 pyramidal cells or DG granule cells were selected for recording based on Soma shape using video microscopy under near infrared differential interference contrast illumination. Cells were visualised on a monitor (Panasonic, UK) using an upright microscope (Leica, Germany) under near infrared differential interference contrast (DIC). Images were enhanced using a camera control unit (Hamamatsu, Japan). Cells were further characterized by their electrophysiological properties obtained from injecting a series of 500 m depolarizing and hyperpolarizing current pulses. Recorded cells were filled with biocytin-dye and neurons were further identified based on their gross morphology (see below).

Spontaneous postsynaptic potentials were recorded from passive membrane responses as mixed spontaneous excitatory postsynaptic potentials (sEPSPs) and spontaneous inhibitory postsynaptic potentials (sIPSPs) at resting membrane potential, and were collected in 60 s frame samples, repeated at 0.33 Hz. The reversal potential of inhibitory events mediated by GABA_A_ receptors was approx.—7 mV. Recordings were carried out under the current clamp mode of operation (NPI SEC 05LX amplifier; NPI electronics, Germany), low pass filtered at 2 KHz and digitized at 5 KHz using a CED 1401 interface (Cambridge Electronic Design, UK). Current-clamp mode allowed for the recording of the intrinsic biophysical properties of the neurons and the natural synaptic voltages to be measured, cells with stable membrane potentials were selected for pharmacological experiments. Input resistance was monitored throughout experiments by means of a hyperpolarizing current step (-10 pA, 10 m). The input resistance was determined from voltage changes in response to hyperpolarizing current steps (-25 pA, 500 m) and calculated from the steady state voltage change. Signal (Cambridge Electronic Design, UK) was used to acquire recordings and generate current steps. The average amplitudes of spontaneous events and their frequency was measured manually from single sweep data sets of 60 s recordings, including a total sweep range of 30–50 frames (*i.e.,* 30–50 min of recording), synaptic noise was taken as ± 0.15mV from baseline, for example values above +0.15mV was considered as synaptic events.

For *in vitro* pharmacological studies, the GABA_A_ receptor antagonist, bicuculline (100 μM, Tocris Bioscience, UK), SNAP5114 (50 μM, Tocris Bioscience, UK), GAT3/4 inhibitor, were bath-applied. Different sub-sets of principal cells were used for each pharmacological protocols, where subsequent addition of the drug was not performed. Drug concentrations were within the range of their reported biological activity with efficacy and in line with previous *in vitro* studies (Wu et al., 2014). Average data points after drug application were obtained after steady-state responses were attained with the drugs, which was ∼15–20 min after onset of the bath-application. Changes in membrane potential caused by drug application were allowed to reach a steady state after ∼15–20 min, and then manually taken to resting membrane potential values to record the spontaneous events to compare the changes to control condition.

### 2.5 Neuroanatomical Procedures and Analysis

#### 2.5.1 Recovery of Biocytin-Labelled Cells Post-Electrophysiological Recordings

After electrophysiological recordings with pharmacological protocols, the slices were fixed in 4% paraformaldehyde and 0.1% glutaraldehyde in 0.1M phosphate buffer for 24 h, embedded in 6% gelatine then re-sectioned at 70 μm. For fluorescence labelling, the sections were permeated using 0.1% Triton X-100 and incubated with Streptavidin-Alexa 488 conjugate (Thermofisher, United States ) for 48 h followed by image acquisition, via confocal laser scanning microscope (LSM 880 Zeiss, Germany). After image acquisition the sections were washed and incubated in avidin-biotin complex (ABC) overnight at 4°C, followed by the 3-3-diaminobenzidine (DAB) staining. Recovered cells were reconstructed manually from consecutive slices at ×100 objective under a Leica DMR microscope with an attached drawing tube.

### 2.6 Immunohistochemical Procedures and Analysis

Ventral hippocampal coronal slices were sectioned at 70 µm thickness using a vibratome (Leica, Germany) from the same region of the DG and CA1 in reference to mouse brain atlases. The brain sections were incubated in 0.1M phosphate buffer solution (PBS) for 24 h on a microplate shaker (VWR, UK). Sections were permeated using 0.3% tris-buffered saline and Triton (TBS-T) solution. This was followed by 1% H_2_O_2_ incubation at room temperature, prepared using 30% stock solution (Sigma-Aldrich, United States) and diluted with deionised water (Sigma-Aldrich, United States). Sections were subsequently washed using TBS-T solution before the blocking procedure using 20% animal serum in PBS. Incubation in primary antibodies was performed for 48 h at 4°C, and subsequent incaution in secondary antibodies was for 3 h at room temperature (Table 2). Following incubation in secondary antibodies, the sections were counterstained using DAPI (1:1000 dilution in H_2_O, Sigma-Aldrich, United States) and mounted with Vectashield (Vector Laboratories, UK). For immunoperoxidase analysis, the slices were incubated in avidin-biotin-horseradish peroxidase complex (Vector Laboratories, UK) solution, processed with DAB, and subsequently dehydrated and mounted (Khan et al., 2018).

**TABLE 2:** List of antibodies and dilutions used in this study.

Primary Antibodies					
Company	Antibody Target	Species	Dilution with TBS-T	Catalog Number	Country
Agilent Technologies	GFAP	Rat	1:1000	13–0300	United States
Merck Millipore	GAD67	Mouse	1:1000	MAB5406	United States
Abcam	GAT3/4	Rabbit	1:100	ab431	United Kingdom
**Secondary Antibodies**					
**Company**	**Antibody Name**	**Targeted Species**	**Dilution with TBS-T**	**Catalog Number**	**Country**
Molecular Probes (now Invitrogen)	Alexa 568	Rat	1:500	A-11077	United States
Abcam	Alexa 488	Rabbit	1:500	ab150077	United Kingdom
Abcam	Alexa 488	Rat	1:500	ab150165	United Kingdom
Sigma-Aldrich	FITC	Mouse	1:200	F2653	United States
Invitrogen	Texas Red	Rabbit	1:750	T2767	United States
Vector laboratories	Biotinylated anti-rabbit	Rabbit	1:500	BA-1000	United States

Human slices (Table 1) followed a similar procedure to mouse brain sections with permeation using TBS-T solution and H_2_O_2_, followed by blocking procedures using 20% animal serum diluted in PBS. The slices were incubated with primary and then subsequently secondary antibody solutions in accordance with dilutions in Table 2 below, before being counterstained with DAPI (1:1000 dilution in H_2_O, Sigma-Aldrich, United States ).

### 2.7 Confocal Microscopy

From each brain section, an average of two Z-stacks at ×20 and ×63 objective were taken using the Zeiss LSM880 confocal microscope in unison with the Zeiss Zen Black imaging software from the DG and CA1. Regions of interest (ROI), CA1 (including stratum oriens, stratum pyramidale and stratum lacunosum) and DG (including the molecular layer, granule cell layer and polymorphic layer), were located using the manual joystick through the ×20 objective lens by systematically searching the slice and consistent evaluation of location in reference to appropriate mouse and human atlases. Z stack images were taken at a resolution of 1024x1024 pixels with 12–14 Z steps through the depth of the slice and with application of appropriate filters to complement secondary antibody fluorescence: DAPI (405λm), FITC/Alexa 488 (488λm), Texas Red/Alexa 568 (561/594λm) and Alexa 647 (640λm).

Single-blinded image analysis was undertaken using the ImageJ software using an automated macro. The Z-stack images were split into their constituent colour channels. Following this, all astrocytes in a given image were selected through the Huang auto thresholding method in the ImageJ software, to demarcate signal from background and produce the ROI (Huang and Wang, 1994). Integrated Density (mean intensity of fluorescence multiplied by area) was calculated for each ROI in the in the x20 Z-stack images and an average taken, with markers such as GAD67 and GAT3/4 measured in astrocyte areas demarcated by GFAP staining using the ROI produced.

### 2.8 Statistics

The statistical analysis was performed using GraphPad Prism version 9.0 for Windows and Microsoft Excel. Based on the differences observed between control and diseased data sets obtained in our preliminary studies, an n ≥ 5 was ideal for this study in order to reveal a statistical difference of >80% power assuming a 5% significance level and a two-sided test for both electrophysiology and neuroanatomy experiments.

All figures displaying error bars represent ± the standard error of the mean. The *“n”* is given as the number of observations and is equal to the number of animals used or human patients, unless otherwise stated. For all statistical tests performed, a 95% confidence interval was used (*p* ≤ .05).

Various statistical tests were performed depending on the parameters used and each figure legends detail the specific statistical test used. A two-way analysis of aariance (ANOVA) corrected for multiple comparisons was used to indicate the presence of significance in neuroanatomical and pharmacological experiments between genotypes or comparisons within a genotype.

## 3 Results

Neuroanatomical studies combined with somatic whole-cell patch recordings were performed on CA1 and DG excitatory cells from 12 to 16 month-old wild-type and *APP* ^*NL-F/NL-F*^ knock-in mouse model. Comparative studies using human hippocampal post-mortem brain tissue were also performed for the neuroanatomical studies.

### 3.1 Elevated Reactive Astrocytes Correlated With Increased Enzyme for GABA and GAT3/4 Levels in AD

**FIGURE 1:**
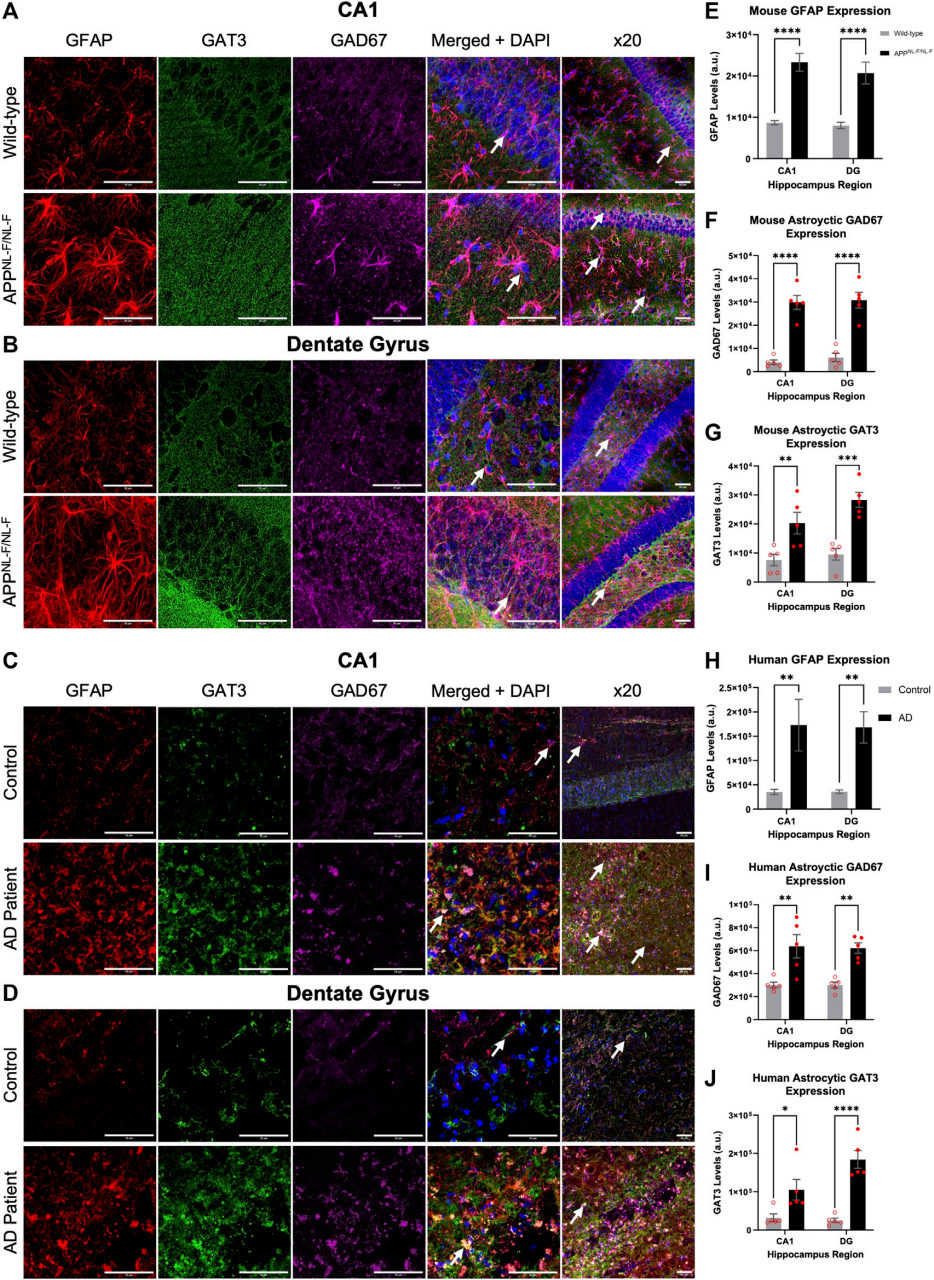
Reactive astrocytes express increased levels of GAT3/4 and GAD67 in AD. **(A–D)** Z-stack images of the CA1 and DG regions of mice show increased Gram-positive astrocytes express increased levels of the astrocyte-specific GABA transporter 3/4 (GAT3/4) colocalised with GAD67 (GABA-producing enzyme) in the *APP* ^*NL-F/NL-F*^ mouse model of AD compared to age-matched wild-type animals. This finding was also consistent in post-mortem brain tissue of AD patients when compared to age-matched controls. White arrows show examples of colocalization at ×63 and ×20 magnification (scale bar = 50 μm). **(E–J)** Graphs show data of astrocytic GFAP, GAD67 and GAT3/4 expression in CA1 and DG, respectively, in mice and human post-mortem brain tissue. The data suggest an increased expression of GFAP, GAD67 and GAT3/4 levels in both, CA1 and DG regions of the AD model as well as in the AD human patient group compared to their control counterparts (*p ≤ .05, **p ≤ .01, ***p ≤ .001, ****p ≤ .0001; Two-way ANOVA with Šidák’s post-hoc multiple comparisons test).

GFAP is a widely used marker of reactive astrocytes, effectively labelling both astrocytic branches and processes in the brain regions investigated here, such as the CA1 and DG of the hippocampus (Zhang et al., 2019). To measure the alteration of the GABA content within astrocytes, we used GAD67 labelling, an enzyme responsible for catalysing the conversion of glutamate to GABA (see methods). In addition, we also stained for astrocyte specific GABA transporter, GAT3/4, to investigate the anatomical changes of astrocytes in AD. Table 3 shows data from all neuroanatomy experiments. Figure 1 illustrates the results from the analysis of immunofluorescence staining (GFAP, GAD67 and GAT3/4) from mouse and human brain slices which included the CA1 and DG regions of the hippocampus (see also Table 3).

**TABLE 3:** Table gives actual values of all neuroanatomy data. All values are stated as mean ± SEM. * denotes significant difference (Two-way ANOVA with Šidák’s post-hoc multiple comparisons test within a genotype, p ≤ .05) between wild-type and APP ^*NL-F/NL-F*^ *mice and human control and human AD cases.*

Tissue	Marker	Region	Wild-type Mean and SEM	APP^NL−F/NL−F^ Mean and SEM
Mouse	GFAP	CA1	8688.82 ± 503.37*	23304.28 ± 2133.60
		DG	8048.76 ± 751.05*	20712.32 ± 2630.37
	GAD67	CA1	4044.04 ± 957.70*	29846.41 ± 3031.23
		DG	6150.76 ± 1797.84*	30769.63 ± 3400.83
	GAT3	CA1	7630.53 ± 1949.88*	20273.58 ± 3730.67
		DG	9533.01 ± 1991.68*	28260.11 ± 2576.60
Human Post-Morten	GFAP	CA1	35322.72 ± 5372.42*	172,552.25 ± 53059.32
		DG	35858.81 ± 3604.18*	168,141.18 ± 32198.47
	GAD67	CA1	30107.36 ± 2491.94*	63793.49 ± 10124.91
		DG	30080.54 ± 2637.88*	62207.25 ± 4578.53
	GAT3	CA1	32237.57 ± 9879.24*	105,136.08 ± 27010.54
		DG	25161.01 ± 5716.40*	183,891.46 ± 23122.87

Quantitively, the results showed a significant increase in the levels of GFAP in the *APP* ^*NL-F/NL-F*^ AD mouse model when compared to age-matched wild-type mice (Figure 1A). The average integrated density for GFAP increased significantly by, 168.21 ± 15.40% in the CA1 region and similarly in the DG region by, 157.34 ± 19.98%. These results were mimicked in the CA1 and DG of human AD patients, when compared to the human control patient group (Figure 1C). Human GFAP levels increased significantly by, 338.50 ± 119.46% in CA1, and by 368.90 ± 70.64% in the DG. The results of the two-way ANOVA revealed a main effect of genotype, but not of the brain area and no interaction among the two factors analysed. The Šidák’s post-hoc multiple comparisons test showed a strong statistical difference among the expression of GFAP in AD tissue (n = 12, ****p ≤ .0001, for mouse study and n = 10, **p ≤ .01 for human study).

GAD67 is expressed by neurons and astrocytes and the overall levels of GAD67 increased in AD tissue. Here we focused on analysing the GAD67 levels in reactive astrocytes, Šidák’s post-hoc multiple comparisons test showed a statistically significant difference between the genotypes, with no significant regional difference within or between the genotypes. There was an increase in GAD67 in astrocytes AD tissue, indicative of increased GABA levels in reactive astrocytes of CA1 and DG of the *APP* ^*NL-F/NL-F*^ mouse model when compared to wild-type control mice (Figure 1F) by, 638.04 ± 64.80% in CA1, and by, 400.26 ± 44.24% in DG (n = 5, ****p ≤ .0001). Similarly, average levels of GAD67, specifically in astrocytes, significantly increased (Figure 1I) in post-mortem brains of Alzheimer’s patients by, 111.89 ± 17.76%*,* and 106.80 ± 7.86% in the CA1 and DG, respectively, when compared to age-matched CA1 and DG of control human patients (n = 5, **p ≤ .01).

To understand astrocyte mediated shutting of GABA and its effects on GABA homeostasis, we analysed the levels of the GAT3/4 GABA transporter within astrocytes. Here, Šidák’s post-hoc multiple comparisons test showed a statistical difference between the expression of GAT3/4 between genotypes, which was markedly increased in *APP* ^*NL-F/NL-F*^ mice when compared to age-matched wild-type control mice by, 165 ± 30.49% and 196.44 ± 17.91 in CA1 and DG, respectively (n = 5, **p ≤ .01), (Figure 1G). In comparison, GAT3/4 levels also increased in AD patients compared to the expression levels in control human tissue by, 226.13 ± 58.09% and 630.86 ± 79.33% in CA1 and DG, respectively (n = 5, **p ≤ .01), (Figure 1J). The results of the two-way ANOVA revealed no interaction among genotype and brin region.

### 3.2 Higher Levels of Tonic Inhibition Observed in CA1 and DG of the *APP* ^*NL-F/NL-F*^ AD Model

**FIGURE 2:**
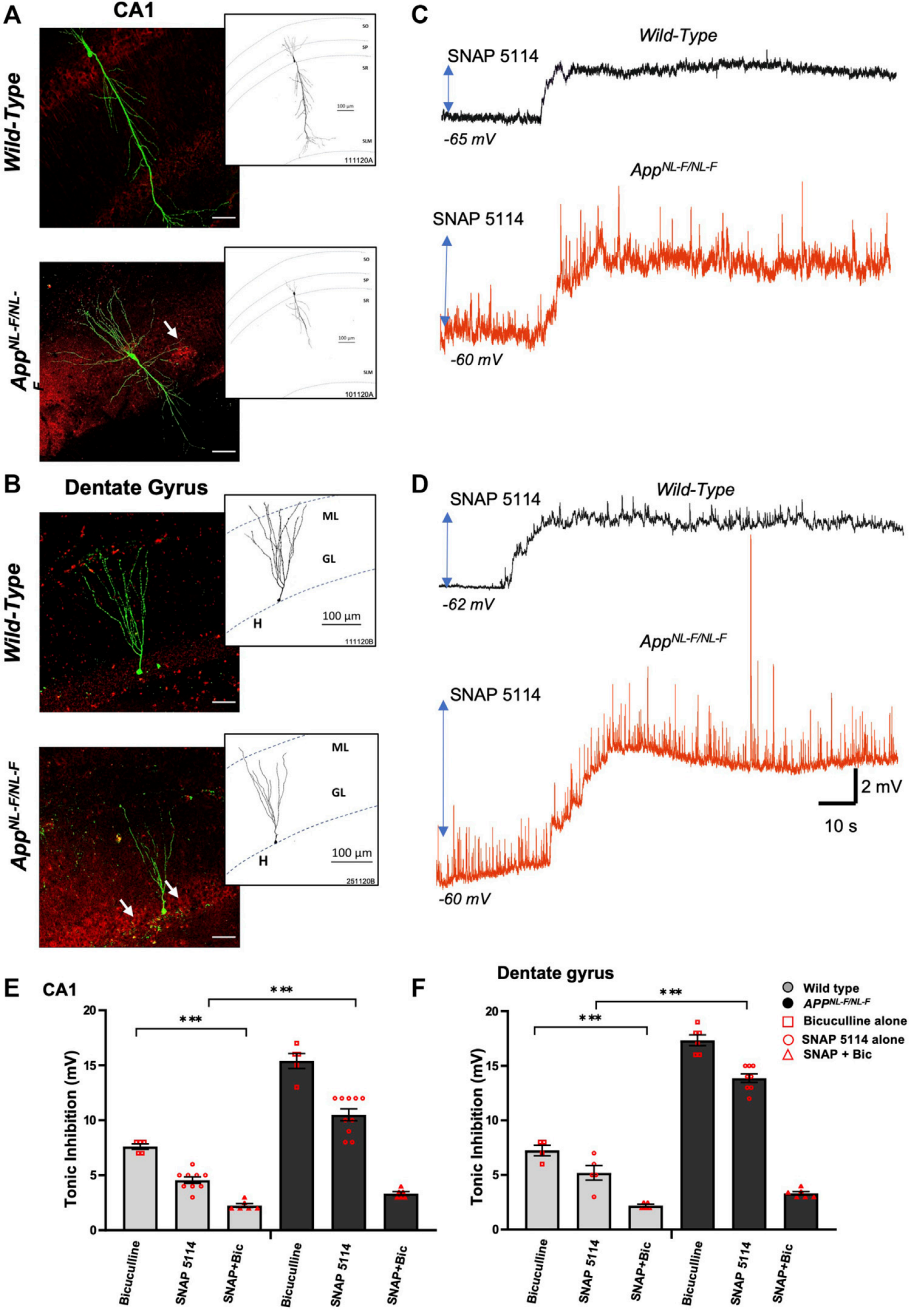
Higher tonic inhibition observed in CA1 and DG of *APP* ^*NL-F/NL-F*^ mice. **(A,B)** Double immunofluorescence performed on whole-cell recorded, biocytin-labelled cells in CA1 and DG with streptavidin (Alexa 488, green) co-labelled with A*β* (Texas red, red) in wild-type and *APP* ^*NL-F/NL-F*^ mice (scale bar 50 µm). The white arrows show A*β* accumulation/plaques which were expressed in higher levels in the AD model. The inserts show reconstructed examples of biocytin-labelled pyramidal and granule cells in control and AD mice, which indicated a reduced dendritic complexity in both types of neurons in the AD model. **(C,D)** Whole-cell electrophysiological recordings from CA1 pyramidal cells and DG granule cells in control conditions (black trace) and after bath-application of the GAT3/4 antagonist, SNAP 5114 (50 µM) (red traces), which depolarised cell membranes, suggesting tonic inhibition under the control of GAT3/4 in all cells recorded. All whole-cell recordings in AD tissue were in close proximity to plaques. **(E,F)** Illustrates the % change in tonic inhibition after bath-application of bicuculline alone in one group of cells, and bath application of SNAP 5114 alone with subsequent bath application of SNAP + bicuculline in a separate group of cells (100 µM) in CA1 and DG principal cells from wild-type and *APP* ^*NL-F/NL-F*^ mice (Two-way ANOVA with Tukey’s post-hoc multiple comparisons test, *p ≤ .05, **p ≤ .01, ***p ≤ .001, ****p ≤.0001)*.*

Whole-cell electrophysiological recordings from principal neurons in either CA1 (Figure 2A) or DG (Figure 2B), revealed a background, tonic inhibition mediated via GAT3/4 in both CA1 and DG of wild-type and *APP* ^*NL-F/NL-F*^ mice. Table 4 shows data from electrophysiology experiments. Figures 2C,D and E-F illustrates the experiments performed to show the tonic inhibition revealed by blockade of GAT3/4 with a selective inhibitor, using SNAP-5114 (50 μM), that altered the resting membrane potential (depolarisation) of all cells recorded. These effects were seen within 20–30 min of bath-application of the drug and suggests that the astrocytic GAT3/4 contributed to the tonic inhibition after acute treatment with the inhibitor.

**TABLE 4:** Table gives actual values of all electrophysiology data. All values are stated as mean ± SEM. *denotes significant difference (p ≤ .05) between wild-type control and experimental values. **denotes significant difference (p ≤ .05) between wild-type and APP ^*NL-F/NL-F*^ *values. A two-way ANOVA was performed corrected for multiple comparisons (α = 0.05), with a post-hoc Tukey’s test.*

	DG		CA1	
sEPSP frequency (Hz)				
	**Control**	**SNAP 5114**	**Control**	**SNAP 5114**
Wild-type	1.79 ± 0.17 (*n = 5*)	2.90 ± 0.29 (*n = 5*) *	2.44 ± 0.15 (*n = 9*)	4.09 ± 0.20 (*n = 9*) *
*APP* ^*NL-F/NL-F*^	3.60 ± 0.16 (*n = 8*) **	4.50 ± 0.16 (*n = 6*) **	3.40 ± 0.13 (*n = 7*) **	4.66 ± 0.17 (*n = 6*)
** sEPSP amplitude (mV)**				
Wild-type	0.89 ± 0.11 (*n = 5*)	1.60 ± 0.18 (*n = 5*) *	0.81 ± 0.07 (*n = 9*)	1.39 ± 0.10 (*n = 10*) *
*APP* ^*NL-F/NL-F*^	1.45 ± 0.11 (*n = 8*)	2.20 ± 0.19 (*n = 6*)	1.46 ± 0.06 (*n = 7*) **	2.43 ± 0.09 (*n = 6*) **
** sIPSP frequency (Hz)**				
Wild-type	1.46 ± 0.18 (*n = 5*)	2.42 ± 0.28 (*n = 5*) *	1.75 ± 0.15 (*n = 9*)	3.06 ± 0.23 (*n = 9*) *
*APP* ^*NL-F/NL-F*^	1.00 ± 0.10 (*n = 8*)	2.67 ± 0.25 (*n = 7*)	0.94 ± 0.09 (*n = 8*) **	2.56 ± 0.27 (*n = 7*)
** sIPSP amplitude (mV)**				
Wild-type	0.70 ± 0.07 (*n = 5*)	1.40 ± 0.11 (*n = 5*) *	0.92 ± 0.12 (*n = 9*)	1.58 ± 0.14 (*n = 9*) *
*APP* ^*NL-F/NL-F*^	0.51 ± 0.04 (*n = 8*)	1.32 ± 0.24 (*n = 6*)	0.54 ± 0.07 (*n = 8*)	1.12 ± 0.13 (*n = 7*)
** Resting membrane potential (mV)**				
Wild-type	-72.00 ± 1.00 (*n = 6*)	-66.84 ± 1.45 (*n = 6*) *	-66.00 ± 0.82 (*n = 8*)	-61.62 ± 0.80 (*n = 8*) *
*APP* ^*NL-F/NL-F*^	-64.00 ± 0.78 (*n = 8*) **	-50.12 ± 0.81 (*n = 8*) **	-62.00 ± 0.53 (*n = 10*) **	-52.70 ± 0.98 (*n = 10*) **
**Tonic inhibition amplitude (mV)**				
DG				
	SNAP 5114	Bicuculline	SNAP 5114 + Bicuculline	
Wild-type	5.20 ± 0.66 (*n = 5*)	7.20 ± 0.48 (*n = 4*)	2.20 ± 0.12 (*n = 5*) *	
*APP* ^*NL-F/NL-F*^	13.90 ± 0.40 (*n = 8*) **	17.30 ± 0.50 (*n = 6*) **	3.30 ± 0.16 (*n = 6*)	
CA1				
	SNAP 5114	Bicuculline	SNAP 5114 + Bicuculline	
Wild-type	4.50 ± 0.29 (*n = 9*)	7.60 ± 0.24 (*n = 5*) *	2.25 ± 0.17 (*n = 6*) *	
*APP* ^*NL-F/NL-F*^	10.50 ± 0.53 (*n = 10*) **	15.40 ± 0.68 (*n = 5*) **	3.34 ± 0.16 (*n = 6*)	

To investigate the involvement of GABA and whether the tonic inhibition was higher in *APP* ^*NL-F/NL-F*^ mice, we bath applied GABA_A_ receptor antagonist, bicuculline (100 µM), to a subset of neurons (Figures 2G,H), which resulted in a change in the resting membrane potential that induced an increase in depolarization in all principal cells and an increase in the input resistance recorded in both wild-type and AD tissue.

The results of the Two-way ANOVA revealed a main effect of genotype and drug effect with interaction between both factors. The Tukey’s post-hoc multiple comparisons test, revealed a significantly greater change in the AD tissue with bicuculline by, 102.63 ± 0.34% and 134.23 ± 0.28% in CA1 and DG respectively (n = 5, ***p ≤ .001) compared to neurons recorded in wild-type CA1 and DG principal cells, similar findings were reported previously by others (Wu et al., 2014). Subsequent bath addition of SNAP-5114 after bicuculline did not affect depolarization any further in CA1 and DG, suggesting that the effects of SNAP-5114 are mediated by GABA_A_ receptors.

In CA1, bath-application of SNAP-5114 (alone) resulted in a change in the average resting membrane potential of -66 ± 0.82 mV to -61.6 ± 0.80 mV and -62.8 ± 0.53 mV to -52.7 ± 0.97 mV in age-matched wild-type and *APP* ^*NL-F/NL-F*^ mice, respectively (Table 4) (*n = 8 wild type, n = 10 for APP* ^*NL-F/NL-F*^ , **p ≤ .01 for wild-type and ***p ≤ .001 for APP ^*NL-F/NL-F*^). Similarly, in the dentate gyrus, application of SNAP-5114 resulted in membrane depolarization, with a significant change in the average resting membrane potential of -72 ± 1 mV to -66.8 ± 1.4 mV in wild-type animals, and a change of -64 ± 0.77 mV to -50.1 ± 0.81 mV in the *APP* ^*NL-F/NL-F*^ mice (*n* = 6 wild-type, *n = 8 APP* ^*NL-F/NL-F*^ , *p ≤ .05 for wild-type, ***p ≤ .001 for APP ^*NL-F/NL-F*^).

The overall tonic inhibition revealed by blockade of GAT3/4, in CA1 was in the range of 4–6 mV, and 8–12 mV in wild-type and *APP* ^*NL-F/NL-F*^ mice, respectively (Table 4), illustrating an enhanced tonic inhibition in the AD model by 130.5 ± 0.31% (*n = 9* wild-type, *n = 10 APP* ^*NL-F/NL-F*^ , ****p ≤ .0001). Interestingly, the overall tonic inhibition was higher in the dentate gyrus, showing an increase of, 166.8 ± 0.25% in the *APP* ^*NL-F/NL-F*^ mice compared to age-matched wild-type mice (range: 13–15 mV, *n = 5* wild-type, *n = 8 APP* ^*NL-F/NL-F*^ , ****p ≤ .0001).

The involvement of GABA in the total tonic inhibition was revealed by the change of membrane potential after subsequent bath-application of 100 µM bicuculline (in the presence of SNAP 5114) to block all GABA_A_-mediated synaptic events. The subsequent tonic inhibition with bicuculline + SNAP-5114, was in the range of 2–3 mV and 3–4 mV in the CA1 and DG, respectively (see Table 4).

### 3.3 GAT3/4 Exacerbates Excitability in the AD Model

**FIGURE 3:**
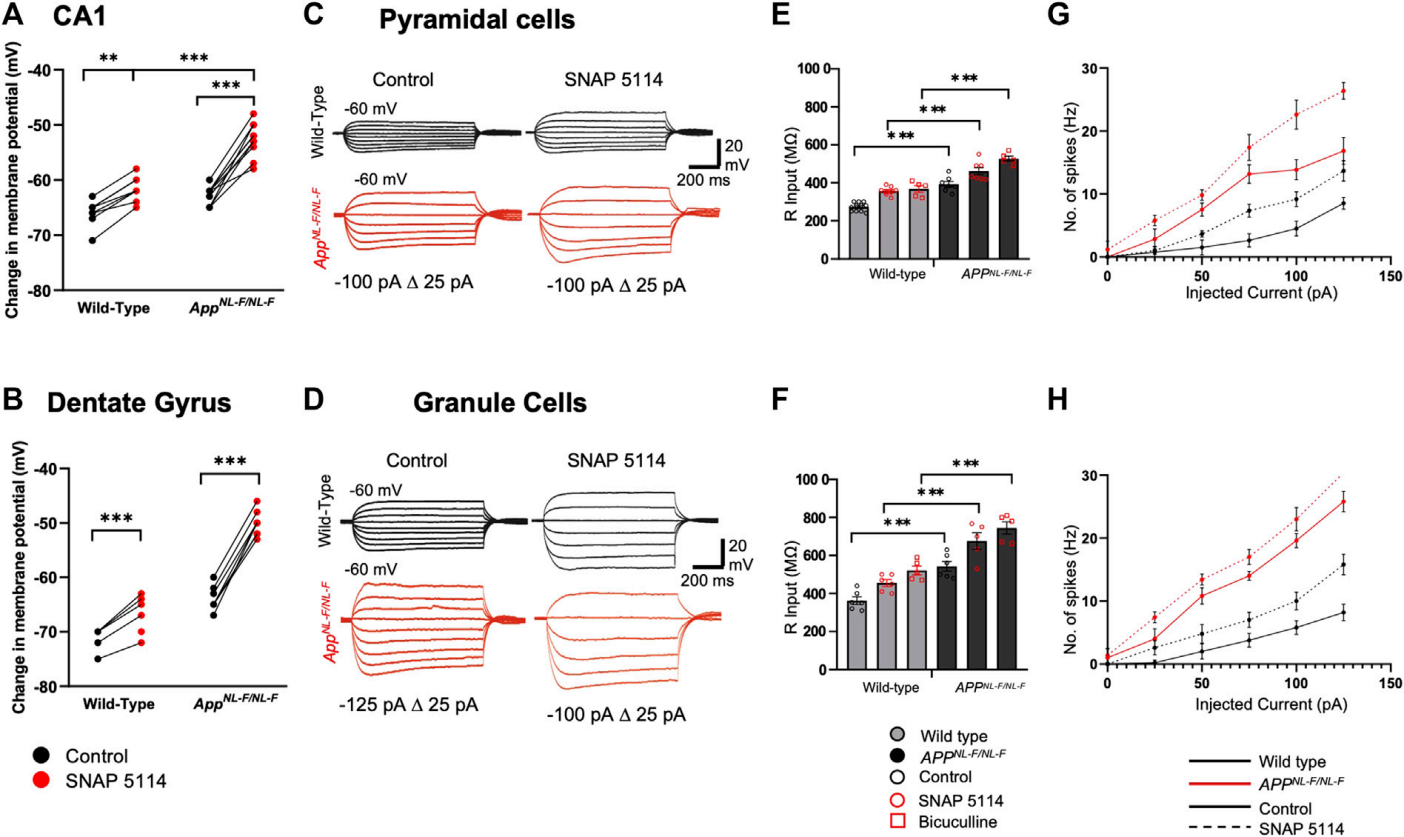
Subthreshold and excitability responses of CA1 and DG principal cells after blockade of tonic inhibition. Whole cell current clamp recordings from CA1 and DG pyramidal cells from wild-type and *APP* ^*NL-F/NL-F*^ mice in control, after bath application with SNAP 5114 or bicuculline. **(A,B)** Graphs detail the change in the resting membrane potential for individual CA1 (upper) and DG (lower) pyramidal cells during bath-application of SNAP 5114 (Two-way ANOVA with Tukey’s multiple comparison test, *p ≤ .05, **p ≤ .01, ***p ≤ .001, ****p ≤.0001). **(C,D)** Voltage responses from CA1 and DG pyramidal cells to 500 m step current injections ranging from −125 to 50 pA in 25 pA increments. For clarity control and SNAP 5114 results are shown. **(E,F)** Input resistance was significantly higher in both CA1 and DG after bath application of SNAP 5114 and bicuculline (*n ≥ 5* for each cohort*,* Two-way ANOVA with post hoc Tukey’s test for multiple comparisons, *p ≤ .05, **p ≤ .01, ***p ≤ .001, ****p ≤ .0001). **(G,H)** Relations between injected current and firing frequency for CA1 and DG pyramidal cells that were more excitable after drug application in wild-type and *APP* ^*NL-F/NL-F*^ mice. The input-output curves displayed a pseudo-linear relationship between no. of spikes generated with increasing current injections in wild-type and the AD model (*n ≥ 5* cells per cohort).

**FIGURE 4:**
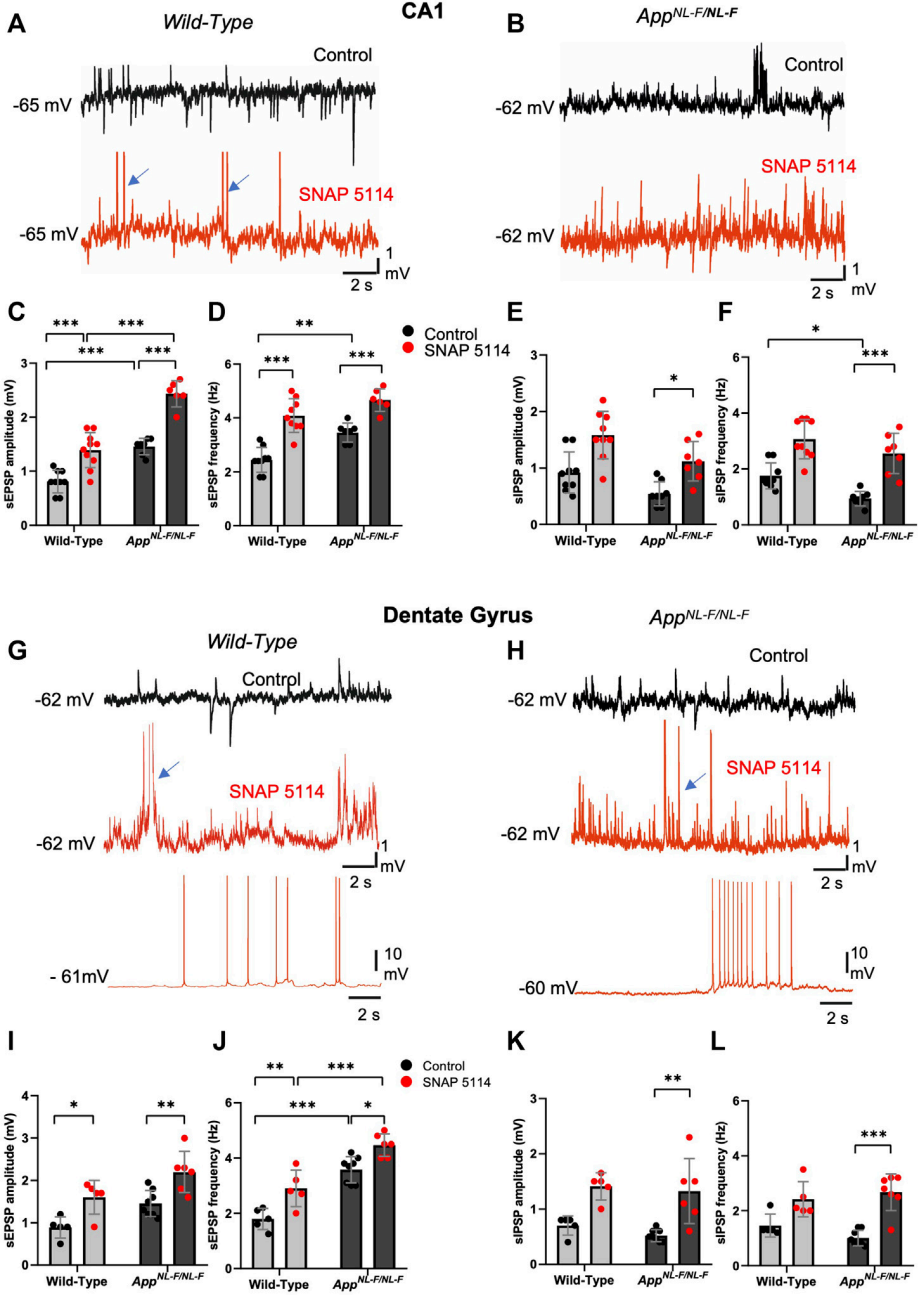
The effect of GAT3/4 on spontaneous synaptic activity. **(A,B** and **G,H)** Whole-cell recordings of CA1 pyramidal cells and DG granule cells showing single sweep traces of spontaneous synaptic events recorded at the cells resting membrane potential, in control (black traces) and after subsequent addition bath-application of SNAP 5114 (red trace) in 12-months old wild-type and *APP* ^*NL-F/NL-F*^ mice. Traces show an increased level of spontaneous excitation, increased sEPSP and action potential firing with SNAP 5114 bath application in both wild-type and *APP* ^*NL-F/NL-F*^ mice. The blue arrows indicate truncated action potentials. **(C–F,I–L)** Graphs show frequency and amplitude of sEPSPs and sIPSPs in each of these regions in wild-type age matched to *APP* ^*NL-F/NL-F*^ mice recorded in control conditions and after subsequent addition of SNAP 5114 (Two-way ANOVA with multiple comparisons test, *p ≤ .05, **p ≤ .01, ***p ≤ .001, ****p ≤ .0001)

To explore the cells excitability after blocked tonic inhibition, the resting membrane potential (RMP), input resistance (R input) and neuronal firing properties that are contributing factors to the differences in excitability (Figure 3) together with the changes in spontaneous synaptic events (Figure 4) were measured.

Figure 3 (A-B) shows RPM of principal cells, which were more depolarised in the *APP* ^*NL-F/NL-F*^ mice in both CA1 and DG, and further depolarized after the bath application of SNAP 5114. The input resistance of the principal cells recorded ranged from, 240 to 300 and 340–600 MΩ under control conditions in CA1 and DG, respectively. As expected, blocking of GABA_A_ receptors with bicuculine resulted in an increase in the input resistance by, 34.57 ± 4.55% in wild-type mice, and 34.49 ± 3.43% in *APP* ^*NL-F/NL-F*^ mice (*n = 5*, ***p ≤ .001) in CA1. Similarly, in DG there was an increase by, 43.95 ± 7.11% in wild-type mice, and 37.08 ± 8.70% in *APP* ^*NL-F/NL-F*^ mice (n = 5, ***p ≤ .001) (Figures 3C–F). Blocking GAT3/4 with SNAP 5114 also significantly increased input resistance by, 30.67 ± 1.82% (n = 8, ***p ≤ .001), and 17.64 ± 2.84% (n = 7, **p ≤ .01) of control conditions in wild-type and *APP* ^*NL-F/NL-F*^ in CA1, respectively. Similarly, in DG, with bath application of SNAP 5114, there was an increase in input resistance by, 25.57 ± 3.59% and 24.53 ± 8.53% of control conditions in wild-type and *APP* ^*NL-F/NL-F*^, respectively (n = 6, *p ≤ .05), shown in Figure 3 (C-F). These changes were associated with increased firing frequency of pyramidal cells after bath application of SNAP 5114 and bicuculline in both genotypes and regions studied (Figures 3G,H).

Spontaneous synaptic events recorded from principal cells displayed hyperactivity in the *APP* ^*NL-F/NL-F*^ mice in contrast to age-matched wild type mice; this activity was further exacerbated by bath-application of the GAT3/4 inhibitor SNAP-5114, which significantly increased the amplitude and frequency of the sEPSPs. The EPSP amplitude was significantly increased in both CA1 and DG of *APP* ^*NL-F/NL-F*^ mice by, 66.9 ± 0.14% and 51 ± 0.19% (n = 6, **p ≤ .01) of control values, respectively. Furthermore, the frequency of sEPSPs was significantly increased in both hippocampal regions of *APP* ^*NL-F/NL-F*^ mice by, 35 ± 0.27% (n = 7, ***p ≤ .001) and 24.9 ± 0.26% (n = 6, *p ≤ .05) in CA1 and DG, respectively. This illustrates a further enhancement of the aberrant hyperactivity in the AD model (Figure 4 red traces). The increase in hyperexcitability as a result of bath-application of SNAP-5114 is also indicated by the change in the cells subthreshold values, and membrane potential to firing threshold in the AD model (Figure 3).

The sIPSP amplitude and frequency also increased with bath application of SNAP-5114 in CA1 and DG, but only significant differences were seen in the *APP* ^*NL-F/NL-F*^ mice in both CA1 and DG (n = 9, ***p ≤ 0.001) (Figure 3 E-F and K-L, see Table 4 for values).

## 4 Discussion

This study reveals novel data concerning the possible role of the GABA transporter GAT3/4 in AD, and provides a mechanistic insight into the pathophysiology of AD in terms of synaptic imbalance governed by astrocyte-specific mechanisms that contribute to altered background tonic inhibition in the first knock-in *APP* ^*NL-F/NL-F*^ mouse model of AD. Our key findings corroborate other studies in the field, which show an enhanced inflammatory response as a result of reactive astrocytes, correlating with an elevated expression of GAD67 and GAT3/4 in hippocampal regions in AD models (Kersanté et al., 2013; Wu et al., 2014; Heneka et al., 2015; Carter et al., 2019). Nevertheless, it should be noted that while GFAP is a key component of most reactive astrocytes, its increase is not necessarily proportional to the level of inflammation especially due to the differences in basal levels of GFAP and physiological responses (Sosunov et al., 2014; Zhang et al., 2019; Escartin et al., 2021).

Our key findings firstly indicate anatomical alterations, including an elevated level of the GAD67 enzyme expressed by astrocytes. This suggests an increase in GABA production in astrocytes, correlated with elevated levels of the astrocyte-specific GAT3/4 in both CA1 and DG regions of the hippocampus in our mouse model of AD. This is consistent with our comparative studies using post-mortem human brain tissue from AD patients. It should be noted that, although some studies suggest GAT3/4 is exclusively expressed in astrocytes (Minelli et al., 1996; Lee et al., 2006), it is conceivable that these receptors are predominantly, but not exclusively expressed on astrocytes. Therefore, in the present study we have measured the expression of GAT3/4 only from astrocyte region as stained by GFAP and performed electrophysiological experiments using GAT3/4 specific pharmacological agent (Dhar et al., 2002).

Secondly, we observed a significant physiological change in synaptic balance in the hippocampus of *APP* ^*NL-F/NL-F*^ mouse model, which showed a higher level of baseline spontaneous synaptic excitation, reduced phasic spontaneous inhibitory events, and an increased background tonic inhibition which was revealed after blocking the GAT3/4 with SNAP-5114. These observations are known collectively as AD-associated hyperexcitation, reported in this study as well as by others (Hazra et al., 2013; Busche and Konnerth, 2016; Ghatak et al., 2019). Previously, using the *APP* ^*NL-F/NL-F*^ mouse model we showed that the AD-associated synaptic imbalance initiates in the entorhinal cortex, an interface for hippocampal-cortical communication, and one of the first regions to be severely affected preceding typical AD pathology. This further spreads the pathology to the CA1 and later to other cortical regions (Khan et al., 2014; Petrache et al., 2019), and corroborates preclinical imaging studies in human patients (Khan et al., 2014). We suggest that the resulting increased synaptic hyperactivity, due to disrupted glutamate levels in various cortical regions, triggers the reactive astrocytes to uptake the excess glutamate, as part of their physiological role in maintaining synaptic homeostasis. This probably leads to the subsequent conversion of the excess glutamate to GABA via the upregulated GAD67 enzyme responsible for catalysing glutamate decarboxylation. However, this is in contrast with some studies that report no significant change in the levels of overall GAD67 or its content within astrocytes using the APP/PS1 mouse model of AD, which differentially expresses A*β*42/Aβ40 expression (Jo et al., 2014) compared to the *APP* ^*NL-F/NL-F*^ mouse model. Furthermore, previous data shows the enhancement of the putrescine metabolic pathway for GABA production as the main mechanism for elevated GABA levels (Héja et al., 2012). Thus, the GAD pathway to produce GABA may be upregulated as an overspill mechanism which functions alongside putrescine metabolism in GABA production, however further research is necessary in knock-in models of AD.

The mechanisms by which GAT3/4 contributes to modulating tonic inhibition are complex and could emulate the multiple roles transporters play in maintaining synaptic homeostasis through interactions of neurons and astrocytes. These mechanisms are likely disrupted in pathological conditions, such as AD. Other studies have also shown the involvement of other GABA transporter (GAT1/2) in pathology such as typical absence seizures in thalamocortical neuronal circuits using genetically modified rat models (Cope et al., 2009). The alternative involvement of other GABA transporters may be due to differential expression and levels of the GABA transporters, especially GAT1 and GAT3 in the CNS. Not only this but often these different transporters may be expressed on different structures such as neuronal membranes and astrocytes respectively (Madsen et al., 2010; Zhou and Danbolt, 2013). Under healthy physiological conditions, astrocyte-specific GAT3/4 plays a role in maintaining the ‘correct’ extracellular environment for neuronal function and tonic inhibition, modulating network behaviour through the uptake of excess GABA from the synaptic environment. In addition, the activity of GAT3 has also been shown to inhibit neuronal glutamate release via the activation of presynaptic adenosine A1 receptors due to a rise in intracellular astrocytic Na^+^ and Ca^2+^ through the Na/Ca exchange, leading to the subsequent release of ATP/Adenosine from the astrocyte (Boddum et al., 2016). Therefore, through such mechanisms, the blockade of GAT3 with SNAP in WT mice may lead to the less significant depolarization seen in our results. Furthermore, the neuropathological role of GABA transporters in astrocytes remains to be fully explored and is further complicated by the conflicting data reported previously. For example, some studies suggest that dampening of GAT3/4 transporter mechanisms results in an increase in tonic inhibition. This is evidenced either via elevating GAT3/4 activity, in a Rett syndrome rodent model, leading to a lowered tonic inhibition (Dong et al., 2020), or reducing the transporter expression, in a Parkinsonian rodent model, leading to an increase in tonic inhibition (Chazalon et al., 2018). These observations are contrary to our results in an AD model which show that the blockade of GAT3/4 results in reduced tonic inhibition in *APP* ^*NL-F/NL-F*^ mice in both the CA1 and DG regions, suggesting that GAT3/4 switches from ‘clearing’ excess extracellular GABA, to extruding GABA. This corroborates with previously published data using the 5xFAD model (Wu et al., 2014).

The differences in these observations could be related to the differential neurological disease models, and a proposed mechanism by which reversal of GAT3/4 function leading to the expulsion of GABA from the astrocytes could be related to AD-associated hyperexcitation in neurons which impact on the intra-astrocyte homeostasis. Thus, the enhanced tonic inhibition in AD may be due to the increased uptake of excess extracellular glutamate (as a direct impact of hyperexcitation) by astrocytes through EAAT1/2 co-transporters resulting in an increase in the intracellular [Na^+^]. This has been shown to lead to a reversal of GAT3/4 channel mechanisms; resulting in the efflux and expulsion of GABA instead of its uptake from the synaptic cleft (Héja et al., 2009, 2012; Wójtowicz et al., 2013). Our hypothesis is also supported by evidence that the blockade of EAAT in astrocytes results in elevated extracellular glutamate levels followed by neuronal death in the hippocampal CA1 and DG regions, suggesting that the elevated tonic inhibition via GAT3/4 serves as a *protective* mechanism in AD (Montiel et al., 2005; Héja et al., 2009). This is also supported by our data showing that blocking the tonic inhibition resulted with an increase in input resistance of principal cells which will raise the neuron’s voltage level quicker, impacting on the resting membrane potential and in turn result in the cell being more readily available to fire action potentials, thus a more excitable state. This synaptic hyperexcitability was evident in both wild-type and AD tissue with bath application of the GAT3/4 blocker, which is not the desired outcome in a system that is altered and in a ‘hyperactive’ state as indicated by the differences in the resting membrane potentials observed between mice cohorts in control conditions. The increase in the amplitude and frequency of the spontaneous and inhibitory events in the present study is probably a direct outcome of the change in membrane potential favouring an increased driving force for GABA through GABA_A_ receptors (Bonin et al., 2007; Herd et al., 2008).

Furthermore, it has been suggested that blocking the upregulated tonic inhibition mediated by α5 subunit-containing GABA_A_ receptors was beneficial in targeting AD, as it favoured an enhancement of long-term potentiation (LTP), a recognised memory parameter (Wu et al., 2014). Our experimental protocol allowed for changes in neuronal membrane and synaptic excitability to be measured, and we consistently observed that blocking GAT3/4 in our AD model resulted in increased firing of principal cells, which could indeed lead to acute enhancement of LTP. Nevertheless, the long-term effects of this change and whether learning or memory are maintained, the pathology of AD halted, or neuronal damage exacerbated by prolonged hyperexcitation, is yet to be determined.

The mechanisms by which tonic inhibition was enhanced through GABA_A_ receptors is an interesting point, as various neuronal sub-types of GABA_A_ receptors have been implicated, including, the α5, α4/6 and δ-subunit-containing GABA_A_ receptors (Caraiscos et al., 2004; Glykys et al., 2008; Lee and Maguire, 2014). It is well documented that the synaptic and extra-synaptic α5 GABA_A_ subunit is “preserved” in AD, as shown in human tissue as well as mouse models of AD (Howell et al., 2000; Petrache et al., 2020). We have also previously shown that this receptor is upregulated in principal cells and interneurons and is responsible for the exacerbation of hyperactivity of principal neurons adding to the spread of aberrant excitation observed in AD brain tissue (Shi et al., 2020). Additionally, it has been shown that the δ subunit which is primarily correlated with the α6 and α4 subunits (Quirk et al., 1995; Jechlinger et al., 1998), is involved in tonic inhibition via slowing of the acute desensitization and recovery rate of GABA-induced currents in mouse fibroblast cells (Saxena and Macdonald, 1994). This was further established in mice where GABA_A_ δ-subunit gene knockout caused convulsive seizures and faster decay rates of sIPSPs (Spigelman et al., 2002). However, the role that these synaptic and extra-synaptic GABA_A_ receptor subunits play in AD pathogenesis is yet to be explored further.

In conclusion, our data are consistent with the hypothesis that AD pathogenesis in the hippocampus is associated with an elevated GABA content in reactive astrocytes, which together with an increased expression of GAT3/4 transporters may lead to an augmented tonic inhibition. Since bath-application of SNAP-5114 in our experiments, exacerbated AD-related synaptic hyperactivity (which is predicted to progress AD pathogenesis), the therapeutic inhibition of the GAT3/4 transporter in AD may be a questionable strategy.

## Data Availability

The raw data supporting the conclusion of this article will be made available by the authors, without undue reservation.

## Abbreviations

A*β*, amyloid-*β*; AD, Alzheimer’s disease; App, *β*-amyloid precursor protein; CA1, Cornu Ammonis; GABA, *γ*-aminobutyric acid; GAD67, glutamate decarboxylase 67; GAT3/4, GABA Transporter-3/4; GFAP, glial fibrillary acidic protein; sEPSPs, spontaneous excitatory postsynaptic potentials; sIPSPs, spontaneous inhibitory postsynaptic potentials.
